# Is this really just “fatigue”? A case series of immune‐related central adrenal insufficiency secondary to immune checkpoint inhibitors

**DOI:** 10.1002/ccr3.1567

**Published:** 2018-05-24

**Authors:** Misako Nagasaka, Nadine Abdallah, Julie Samantray, Ammar Sukari

**Affiliations:** ^1^ Department of Oncology Barbara Ann Karmanos Cancer Institute Wayne State University School of Medicine Detroit MI USA; ^2^ Department of Advanced Medical Innovation St. Marianna University Graduate School of Medicine Kawasaki Kanagawa Japan; ^3^ Department of Medicine Wayne State University Detroit MI USA; ^4^ Department of Endocrinology Wayne State University Detroit MI USA

**Keywords:** fatigue, immune‐related hypophysitis, PD1/L1 checkpoint inhibitor

## Abstract

While immunotherapy with programmed cell death protein 1 (PD1) checkpoint inhibition has shown promising activity against many tumor types, adverse events are common. Hypophysitis is a rare but serious immune‐related event known to occur with anti‐PD1 inhibition. It will become more prevalent as the usage of checkpoint inhibitors increases.

## 1 INTRODUCTION

While immunotherapy targeting the programmed cell death protein 1 (PD1) checkpoint inhibition has shown promising activity against many tumor types, adverse events caused by these agents are common. Central adrenal insufficiency, sometimes referred to as hypophysitis, is a well‐documented and rare but serious immune‐related event known to occur with anti‐PD1 inhibition.

## 2 CASE PRESENTATION

### 2.1 Patient 1

A 61‐year‐old man had originally presented in 2012 with stage IVA oropharyngeal squamous cell carcinoma (SCC). He was treated with surgery followed by chemoradiation. Eighteen months later, he presented with a pancreatic mass and multiple osteolytic lesions. These were proven by biopsy to be metastatic oropharyngeal SCC. He was treated with carboplatin, 5‐fluorouracil, and cetuximab with resolution of the pancreatic mass. On follow‐up CT post 6 months of maintenance cetuximab, he was noted to have an asymptomatic left occipital brain metastasis for which he underwent stereotactic radiation. Three months later, he underwent left occipital craniotomy for relapsed disease. Imaging revealed a new enlarged paratracheal lymphadenopathy, and he was started on nivolumab at a dose of 3 mg/kg. After 3 months, he had a partial response. However, when he presented for day one of cycle 8, he complained of sudden onset fatigue. Brain MRI showed no new lesions. There was no evidence of pituitary inflammation. Blood work revealed a low random cortisol level of 1.8 μg/dL that did not have a satisfactory response to the ACTH stimulation test (Table 1). With ACTH <0.5 pg/mL, primary adrenal insufficiency was ruled out, and he was started on dexamethasone followed by hydrocortisone for grade 2 immune‐related central adrenal insufficiency. Because his fatigue was resolved, he resumed nivolumab treatment 5 weeks later. Restaging scans continue to show no evidence of progression. The patient continues to be on hydrocortisone with no complaints of fatigue.

**Table 1:** Select laboratory values and cosyntropin stimulation test results for all patients

	Patient 1	Patient 2	Patient 3
	61 M	61 M	77 M
Select laboratory values			
Na (mmol/L) [135‐147]	134	129	119
Random cortisol (μg/dL)^a^	1.8	<0.5	1.3
ACTH (pg/mL) [7‐69]	<5	<5	<5
FSH (mIU/mL) [1.4‐18.1]	6.8	11.1	4.9
LH (mIU/mL) [1.5‐9.3]	5.5	10.6	4.4
Testosterone (ng/dL) [105‐540]	108	257	202
Prolactin (ng/mL) [2.1‐17.7]	7.2	10	7.9
TSH (mIU/mL) [0.2‐4.78]	4.8	0.702	7.7
Free T4 (ng/dL) [0.8‐1.8]	0.6	1.0	NA
Cosyntropin stimulation test results			
Baseline cortisol	1.5	<0.5	0.8
30 min	5.9	<0.5	4.7
1 h	7.7	1.7	6.6

a Reference values for random cortisol: 7‐9 am 4.3‐22.4, 3‐5 pm 3.1‐16.7

**Table 2:** Definition of CTCAE grades

Grade	Definition
1	Mild; asymptomatic or mild symptoms; clinical or diagnostic observations only; intervention not indicated
2	Moderate; minimal, local or noninvasive intervention indicated; limiting age‐appropriate instrumental ADL
3	Severe or medically significant but not immediately life‐threatening; hospitalization or prolongation of hospitalization indicated; disabling; limiting self‐care ADL
4	Life‐threatening consequences; urgent intervention indicated
5	Death related to AE

ADL, activities of daily living; AE: adverse events; CTCAE, common terminology criteria for adverse events.

### 2.2 Patient 2

A 61‐year‐old man with oral cavity SCC, who had been originally treated with surgery in 2011 for stage I disease, presented with cervical and left axillary lymphadenopathy after being lost to follow‐up for over 4 years. Biopsy of the lymph nodes revealed metastatic SCC. He was then treated with carboplatin, 5‐FU, and pembrolizumab on a trial. Pembrolizumab was dosed at 200 mg. After 4 cycles, his scans showed a partial response, but the patient presented with fatigue and hypotension to systolic blood pressure of 70 seconds. He was found to have a random cortisol level <0.5 μg/d that did not have a satisfactory response to the ACTH stimulation test (Table 1). His ACTH level was <5 pg/mL. He was diagnosed with immune‐related central adrenal insufficiency and was started on high‐dose dexamethasone and later transitioned to hydrocortisone. Brain MRI did not show any inflammation of the pituitary stalk, and it was without metastatic lesions. Ten days later, his fatigue was resolved, and he resumed treatment with pembrolizumab. He continues to take hydrocortisone.

### 2.3 Patient 3

A 77‐year‐old man with stage IV lung SCC with metastasis to the liver was originally treated with carboplatin and nab‐paclitaxel achieving a partial response. Unfortunately, he was found to have disease progression and was started on nivolumab 3 mg/kg. On subsequent scans, he continued to show stable disease. On day 1 of cycle 8, he complained of profound fatigue. He was found to have a random cortisol level of 1.3 μg/dL that did not have a satisfactory response to the ACTH stimulation test. His ACTH was <5 pg/mL (Table 1). As immune‐related central adrenal insufficiency was suspected, he was started on dexamethasone followed by hydrocortisone, and his symptoms were resolved immediately. The patient opted to discontinue nivolumab. Repeat scans 3 months postdiscontinuation of nivolumab continues to show no evidence of progression. He continues to be asymptomatic on hydrocortisone.

## 3 DISCUSSION

Nivolumab and pembrolizumab are monoclonal antibodies that block the interaction of PD‐1 with its ligands, PD‐L1 and PD‐L2. Immune‐related events such as colitis and pneumonitis are known to occur with anti‐PD1 inhibition. Autoimmune hypophysitis is an immune‐related adverse event most frequently reported with treatment with anti‐CTLA‐4 drugs such as ipilimumab.^1^ While symptoms of diarrhea, abdominal pain, shortness of breath, and cough can help direct clinicians toward colitis and pneumonitis, the presentation of autoimmune central adrenal insufficiency with fatigue and hypotension can be challenging for diagnosis.

The incidence of grade 3‐4 hypophysitis is <1% with single‐agent anti‐PD‐1/PD‐L1 antibody therapy and 7%‐17% in combination studies utilizing anti‐PD‐1/PD‐L1 and another agent such as anti‐CTLA‐4.^2^ In the oncology literature, central adrenal insufficiency is sometimes referred to as hypophysitis. Hypophysitis results in the low‐level release of all or some of the pituitary gland hormones. It is diagnosed by biochemical testing of the pituitary‐hypothalamic (prolactin), pituitary‐thyroid (T4, TSH), pituitary‐gonadal (LH, FSH), and pituitary‐adrenal (ACTH, cortisol) axes. Laboratory findings differentiate hypophysitis and central adrenal insufficiency from primary adrenal insufficiency, which is manifested by low cortisol or inappropriate cortisol stimulation and high ACTH, whereas low ACTH suggests a central cause of adrenal insufficiency. In select cases, radiologic evidence of pituitary inflammation was observed.^3^

Central adrenal insufficiency and hypophysitis can be difficult to diagnose because of its nonspecific symptoms, most typically fatigue. Headaches and weakness are also common. Additional signs or symptoms reflecting the deficient hormone could also be an indicator of this condition. For example, orthostatic hypotension, hyponatremia, and hypoglycemia can be seen in corticotrophin deficiency (ACTH).^4^

Weber et al recommend that all patients receiving checkpoint inhibitors routinely undergo thyroid function studies, complete blood counts, liver function, and metabolic panels at each treatment and at intervals of 6‐12 weeks for the first 6 months after completing treatment. Adrenocorticotropic hormone, cortisol, and testosterone should also be checked in patients who develop fatigue and nonspecific symptoms.^5^

Treatment is based on the replacement of the appropriate hormone deficiency, such as hydrocortisone. Central adrenocortical insufficiency may require lifelong glucocorticoid replacement. In rare scenarios, patients may present with adrenal crisis requiring hospitalization, aggressive intravenous corticosteroid, and fluid and electrolyte replacement.^2^ In most cases of grade 1 and 2 autoimmune hypophysitis, checkpoint inhibitors may be safely resumed; however, a case‐by‐case discussion would be required in grade 3. For grade 4, checkpoint inhibitors should not be resumed.

Our three patients all presented with fatigue. All had low random cortisol levels that prompted further evaluation. They also had undetectable ACTH levels (<5 pg/mL) and baseline low cortisol levels that had insufficient response to ACTH testing. None of these patients had involvement of other anterior pituitary axes. One of the three patients was found to be severely hypotensive and was in critical condition at presentation. All three displayed rapid improvements of their symptoms with steroid management, and two were subsequently resumed on checkpoint inhibitors.

## 4 CONCLUSION

Profound fatigue in patients receiving immune checkpoint inhibitors should be granted further evaluation to exclude autoimmune central adrenal insufficiency and other endocrinopathies. Autoimmune central adrenal insufficiency will likely become more prevalent as the usage of checkpoint inhibitors increases. Early recognition and treatment are imperative in providing quality care and managing potentially life‐threatening consequences.

## DECLARATION

Ethics approval and consent to participate: The need for approval was waived by the Karmanos Protocol Review and Monitoring Committee. Consent for publication: The consent for treatment and publication was obtained from the patients themselves. Availability of data and material: Data sharing was not applicable to this article because no datasets were generated or analyzed during the current study.

## CONFLICT OF INTERESTS

The authors have no actual or potential conflict of interests to disclose. There are no competing interests.

## AUTHORSHIP

MN and AS: contributed to the planning, organization, data collection, and writing of the manuscript. AS: is also the treating oncologist for this case and is the corresponding author for this manuscript. NA and JS: provided critical edits to the manuscript. All authors: the final version of the manuscript was approved by all authors.
